# Association between platelet to lymphocyte ratio and the risk of vertebral fracture in patients with osteoporosis: a systematic review and meta-analysis

**DOI:** 10.3389/fendo.2026.1705468

**Published:** 2026-03-25

**Authors:** Feipeng Hu, Youwen Zhan, Jia Yao, Mengjing Cheng, Jiaxin Wang

**Affiliations:** Pain Rehabilitation, Yongkang Orthopedic Hospital, Yongkang, Zhejiang, China

**Keywords:** meta-analysis, osteoporosis, platelet to lymphocyte ratio, systematic review, vertebral fracture

## Abstract

**Background:**

Vertebral fractures, accounting for 40% of osteoporotic fractures, often lack early clinical symptoms, necessitating improved risk prediction biomarkers. This meta-analysis was the first to evaluate the association between platelet-to-lymphocyte ratio (PLR) and vertebral fracture risk in osteoporosis patients.

**Methods:**

We systematically searched PubMed, Embase, Web of Science, Cochrane, Wanfang and CNKI (up to July 2025). Odds ratio (OR) and standardized mean difference (SMD) with 95% confidence intervals (CIs) were used for the data synthesis of categorical and continuous variables, respectively. Sensitivity analysis was performed to explore the stability of the results and potential sources of heterogeneity. All analyses were performed using Review Manager 5.4 and STATA 15.0.

**Results:**

Seven observational studies were included. Categorical data showed significantly higher vertebral fracture risk in high-PLR groups (OR: 1.02; 95% CI: 1.00, 1.03; *P* = 0.01). Continuous data revealed a significantly higher PLR level in the fracture group compared with the non-fracture group (SMD: 1.78; 95% CI: 0.32, 3.25; *P* = 0.02). No significant publication bias was detected for either categorical or continuous variables. Sensitivity analyses revealed instability in both categorical and continuous outcomes and found that the Di et al., 2024 and Song et al., 2022-II studies might be the main sources of heterogeneity.

**Conclusion:**

Elevated PLR is associated with increased vertebral fracture risk in osteoporosis patients, supporting its role as a predictive inflammatory biomarker. However, heterogeneity in study designs and unstable outcomes highlight the need for a standardized PLR cut-off and larger multinational cohorts to validate its clinical utility.

**Systematic Review Registration:**

https://www.crd.york.ac.uk/PROSPERO/view/CRD420251137240, identifier CRD420251137240.

## 1 Introduction

Osteoporosis, a systemic skeletal disease characterized by osteopenia and bone microarchitecture damage, has become a major global public health challenge (1–3). According to the International Osteoporosis Foundation (IOF), an osteoporosis-related fracture occurs every 3 seconds worldwide, with vertebral fractures accounting for approximately 40% of all osteoporotic fractures, and the risk of secondary fractures being about five times higher than that in patients with non-vertebral fractures (4). Epidemiological studies in China show that the prevalence of vertebral fractures in people over 50 years old is about 15%, but only 20%-30% of vertebral fracture patients have typical clinical symptoms, resulting in a misdiagnosis rate of up to 60% (5, 6). Although the fracture risk assessment tool FRAX is widely used in clinical practice, its predictive sensitivity for vertebral fractures is low, especially in patients with early osteoporosis without other risk factors (7). This suggests the need to explore new biomarkers to improve the existing risk assessment system.

In recent years, the role of chronic low-grade inflammation in the pathological process of osteoporosis has attracted much attention. As a marker of systemic inflammation, the platelet-to-lymphocyte ratio (PLR) has been shown to be closely related to bone metabolism imbalance (8–12). Platelets play a dual regulatory role in the development and progression of osteoporosis. On the one hand, platelets release multiple growth factors, directly promoting the proliferation, differentiation, and migration of osteoblasts and stimulating bone matrix synthesis and angiogenesis, thereby maintaining a dynamic balance between bone formation and bone resorption (13). These factors are crucial for bone metabolism. For example, PDGF enhances the chemotaxis of osteoblasts to sites of bone resorption, accelerating bone defect repair (14). Conversely, decreased TGF-β is associated with delayed bone healing and the risk of osteoporosis (15). Lymphocyte subsets inhibit osteoclast activity by producing IL-4, IL-10, IL-17 and osteoprotein to maintain bone remodeling homeostasis (16). Abnormally elevated PLR indicates both excessive platelet activation, which releases proinflammatory mediators, and impaired immune regulation due to a decrease in lymphocyte numbers, both of which together contribute to the persistence of an inflammatory state in the bone microenvironment (12). This chronic inflammatory microenvironment activates signaling pathways such as nuclear factor κB (NF-κB), stimulates the expression of osteoclast differentiation factor (RANKL), and inhibits osteoblast activity, ultimately leading to decreased bone density and destruction of bone microarchitecture (17). A meta-analysis of 24 studies found that elevated PLR was significantly associated with an increased risk of osteoporosis. For continuous variables, individuals with osteoporosis showed significantly higher levels of PLR compared with non-osteoporotic controls (8). This evidence collectively indicate that PLR may become a new indicator for predicting vertebral fractures by reflecting the inflammatory state of the bone microenvironment.

At present, the predictive efficacy of PLR in osteoporosis-related vertebral fractures remains controversial: a retrospective study of 310 patients diagnosed with osteoporosis did not find a significant association between PLR and the risk of vertebral fractures (18), but another prospective cohort study (n=12722) found that PLR was a significant independent risk factor for osteoporotic vertebral fractures (OR = 1.07, 95%CI 1.06-1.09) (19). This heterogeneity may be due to differences in study design, including the timing of PLR detection (acute inflammatory phase vs. stable phase), fracture diagnostic criteria (radiological diagnosis vs. clinical symptoms), and control of confounding factors (such as history of glucocorticoid use). Therefore, this first systematic review and meta-analysis aims to integrate global observational study evidence and quantitatively evaluate the association between PLR and the risk of vertebral fractures in osteoporotic patients. The results will provide important evidence for improving the risk stratification of osteoporotic fractures and formulating individualized intervention strategies, while opening up new perspectives for the translational application of inflammatory mechanisms in bone metabolism disorders.

## 2 Methods

### 2.1 Literature search

This meta-analysis was performed according to the PRISMA (Preferred Reporting Items for Systematic Reviews and Meta-Analyses) 2020 statement (20) and has been prospectively registered in PROSPERO (CRD420251137240). We conducted a systematic literature search via PubMed, Embase, Web of Science, Cochrane, Wanfang, and CNKI up to July 2025 for studies that evaluated the association between PLR and the risk of vertebral fracture in patients with osteoporosis. We searched the literature using the following terms: “Blood Platelets”, “Lymphocytes”, “Osteoporosis”, and “Fracture”. The detailed search strategies in PubMed are as follows: (((((“Blood Platelets”[Mesh]) OR (((((Blood Platelet) OR (Platelets)) OR (Platelet)) OR (Thrombocytes)) OR (Thrombocyte))) AND ((“Lymphocytes”[Mesh]) OR (((Lymphocyte) OR (Lymphoid Cells)) OR (Lymphoid Cell)))) AND (Ratio)) AND ((“Fractures, Bone”[Mesh]) OR (Fracture))) AND ((“Osteoporosis”[Mesh]) OR (Osteoporoses)). Furthermore, we manually screened the reference lists of all included studies. Two authors retrieved and assessed eligible articles independently. Any differences in literature retrieval were resolved through discussion. The detailed literature search strategy is shown in **Supplementary Table 1**.

### 2.2 Inclusion and exclusion criteria

Articles were eligible when meeting the following standards: (1) study design was a randomized controlled trial, cohort, or case-control; (2) studies were performed in patients with osteoporosis; (3) studies evaluated the association between PLR and the risk of vertebral fracture; (4) at least one outcome (including categorical or continuous variables) was evaluated; (5) complete data to analyze multivariate data of odds ratio (OR) or standardized mean difference (SMD) with 95% confidence interval (CI) were provided. We excluded study protocols, unpublished studies, non-original studies (including letters, comments, abstracts, corrections, and replies), studies without sufficient data, and reviews.

### 2.3 Data abstraction

Data abstraction was conducted by two authors independently. Any differences were resolved by another author. We abstracted the following information from eligible studies: first author name, publication year, study country, study design, research population, sample size, age, gender, PLR cut-off, multivariate analysis OR and mean with standard deviation (SD). If the research data were insufficient, corresponding authors were contacted for full data if available.

### 2.4 Quality evaluation

The Newcastle-Ottawa Scale (NOS) was applied to assess the quality of included cohort studies (21), and studies with 7–9 points were considered as high-quality (22). Studies with NOS scores below 7 were not included for quantitative analysis. Two authors independently assessed the quality of all included studies, and any disagreement was resolved by discussion.

### 2.5 Statistical analysis

Meta-analysis was conducted in Review Manager 5.4.1 edition. OR and SMD with 95% confidence intervals (CIs) were used for the data synthesis of categorical and continuous variables, respectively. The chi-squared (χ²) test (Cochran’s Q) and inconsistency index (I²) were applied to evaluate the heterogeneity of each outcome (23). χ^2^ *P* value less than 0.05 or *I*^2^ more than 50% were regarded as high heterogeneity. The random-effects model was applied to calculate the total OR and SMD for each outcome. Additionally, we conducted sensitivity analysis to assess the influence of each included study on the total OR and SMD for results with three or more studies included. Moreover, we assessed the potential publication bias by performing Egger’s regression test (24) through Stata 15.1 (StataCorp, College Station, TX, USA) for outcomes with three or more studies included. A P value < 0.05 was considered as statistically significant publication bias.

## 3 Results

### 3.1 Literature retrieval and study characteristics

**Figure 1:**
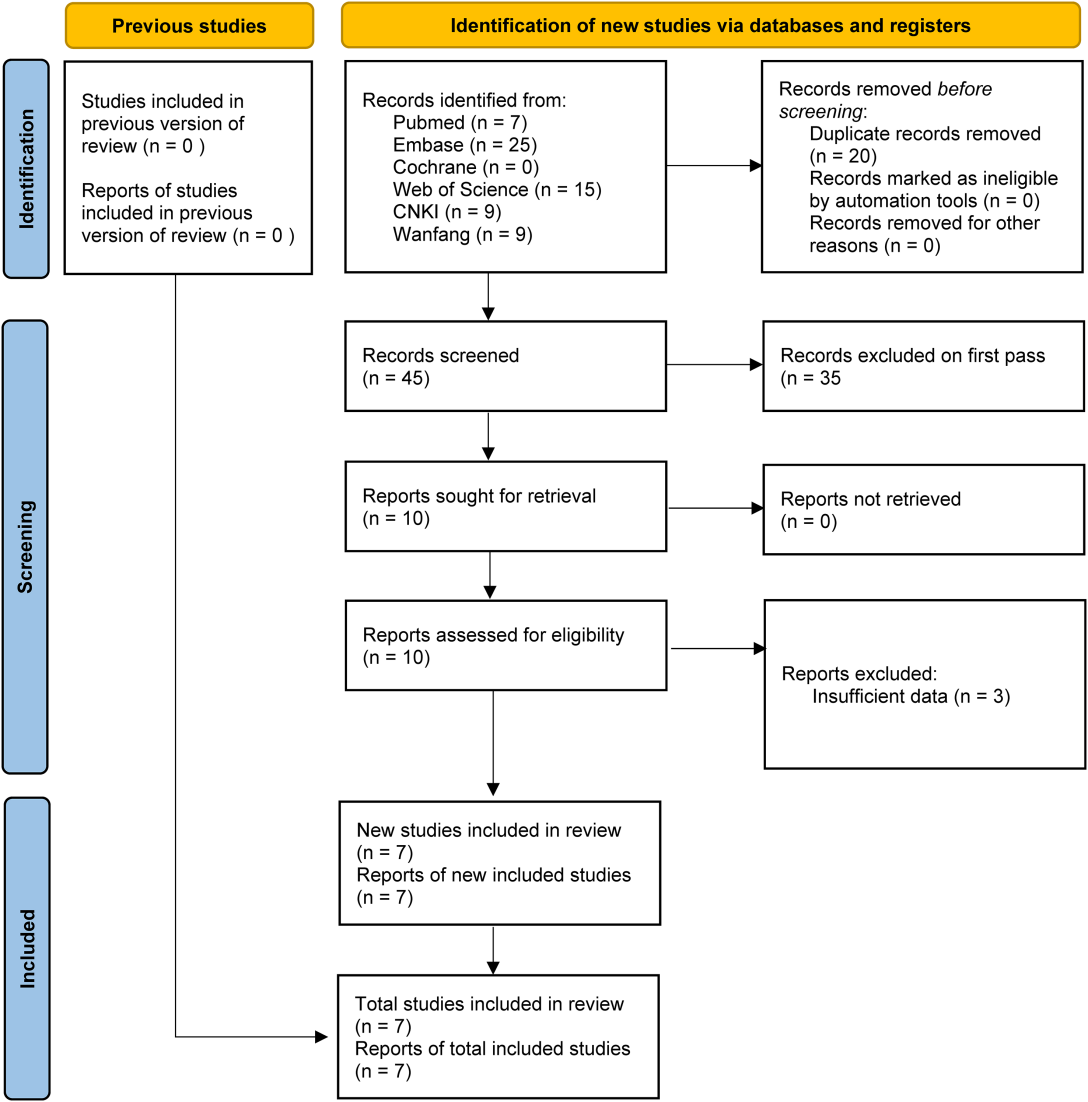
Flowchart of the systematic search and selection process.

**Figure 1** shows the flowchart of the literature retrieval and selection process. A total of 65 related studies in PubMed (n = 7), Embase (n = 25), Web of Science (n = 15), Cochrane (n = 0), Wanfang (n = 9), and CNKI (n = 9) were identified via a systematic literature search. After removing duplicate studies, a total of 45 titles and abstracts were evaluated. Eventually, 7 studies were included for meta-analysis (18, 19, 25–29). **Table 1** presents the characteristics and quality evaluation of each eligible study.

**Table 1:** Characteristics and quality assessment of included studies.

Study	Region	Study design	No. of patients	Gender		Mean/median age	Mean/median BMI	PLR cut-off	NOS score
				Male	Female				
Cai 2023 (25)	China	Cohort	305	65	240	72.14	24.13	133.5	7
Di 2024 (9)	China	Cohort	12722	Data not reported	Data not reported	56.05	Data not reported	Data not reported	7
Duan 2025 (26)	China	Case-control	303	Data not reported	Data not reported	Data not reported	Data not reported	Data not reported	8
Fu 2024 (18)	China	Case-control	310	273	37	67.38	21.91	82.361	7
Gou 2024 (27)	China	Case-control	1281	615	666	68	Data not reported	Data not reported	7
Song 2022-I (28)	China	Cohort	413	0	413	61.9	22.8	Data not reported	7
Song 2022-II (29)	China	Cohort	426	198	228	73.73	22.23	175.04	8

### 3.2 PLR and the risk of vertebral fracture (categorical variables)

**Figure 2:**
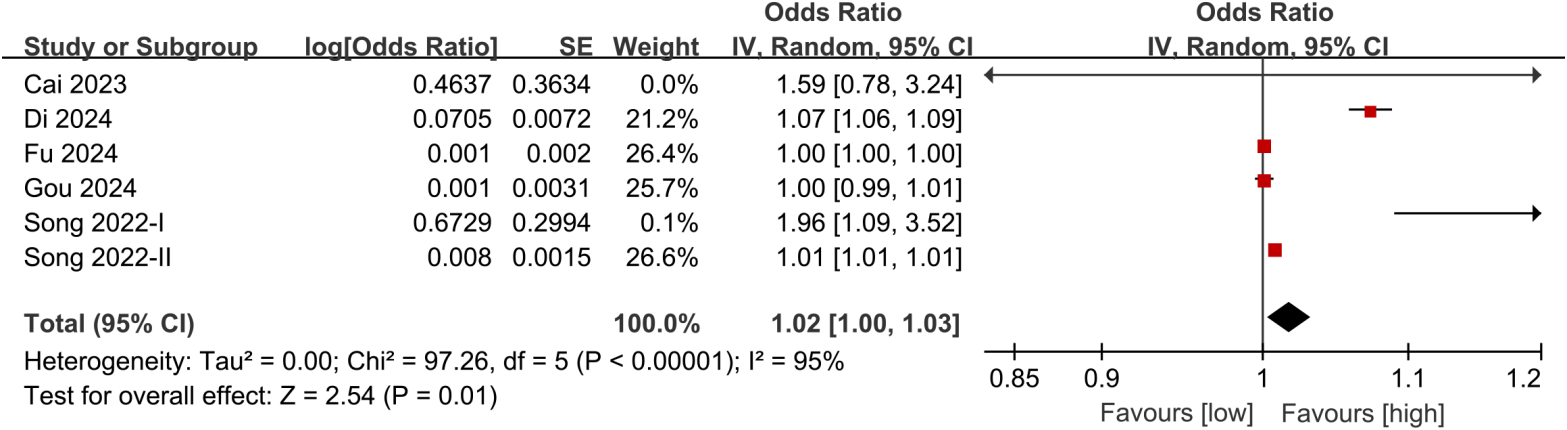
Forest plot of the association between PLR and the risk of vertebral fracture in patients with osteoporosis (categorical variable analysis). This forest plot presents the results of a meta-analysis using a random-effects model to illustrate the relationship between vertebral fracture risk and PLR (high PLR vs. low PLR) as a categorical variable. Six observational studies were included in the plot. Each study is represented by a square indicating its adjusted odds ratio (OR), with the horizontal line representing the 95% confidence interval (95% CI). The size of the square reflects the weight of the study in the pooled analysis. The right-hand data column lists the OR, 95% CI, and weight percentage for each study. The vertical dashed line represents the null line (OR = 1). The diamond symbol represents the pooled effect size, with its width equal to the 95% CI of the pooled OR. The pooled analysis showed that the risk of vertebral fracture was significantly higher in the high PLR group than in the low PLR group (pooled OR = 1.02, 95% CI: 1.00–1.03). The heterogeneity test results (I² = 95%) are reported below the plot, indicating high heterogeneity among the studies.

Results of categorical variables were synthesized from 6 studies, and meta-analysis of multivariate data revealed a significantly higher risk of vertebral fracture in the group with high PLR compared with the group with low PLR (OR: 1.02; 95% CI: 1.00, 1.03; *P* = 0.01). Significant heterogeneity was observed (*I*^2^ = 95%, *P* <0.00001) (**Figure 2**).

### 3.3 PLR and the risk of vertebral fracture (continuous variables)

**Figure 3:**
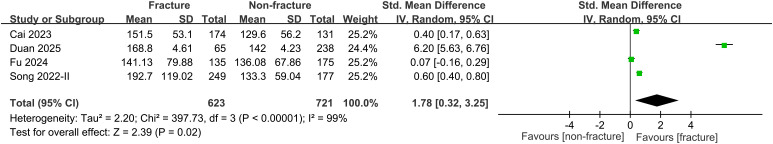
Forest plot comparing PLR levels between the fracture group and the non-fracture group (continuous variable analysis). This forest plot presents the results of a meta-analysis using a random-effects model to show the difference in PLR levels between patients with osteoporotic vertebral fractures and those without fractures, when PLR is considered a continuous variable. Four studies were included in the plot, and effect sizes are expressed as standardized mean difference (SMD) and their 95% confidence intervals. Each study is represented by a square representing its SMD point estimate, with the horizontal line representing the 95% CI, and the square size reflecting its weight. The right-hand data column lists the SMD, 95% CI, and weight for each study. The vertical dashed line is the null line (SMD = 0). The diamond symbol represents the pooled effect size. The pooled analysis showed that PLR levels were significantly higher in patients with vertebral fractures than in those without fractures (pooled SMD = 1.78, 95% CI: 0.32–3.25). The heterogeneity test results (I² = 99%) are reported below the plot, indicating extremely high heterogeneity among the studies.

Results of continuous variables were synthesized from 4 studies, and meta-analysis revealed a significantly higher PLR level in the fracture group compared with the non-fracture group (SMD: 1.78; 95% CI: 0.32, 3.25; *P* = 0.02). Significant heterogeneity was observed (*I*^2^ = 99%, *P* <0.00001) (**Figure 3**).

### 3.4 Publication bias and sensitivity analysis

**Figure 4:**
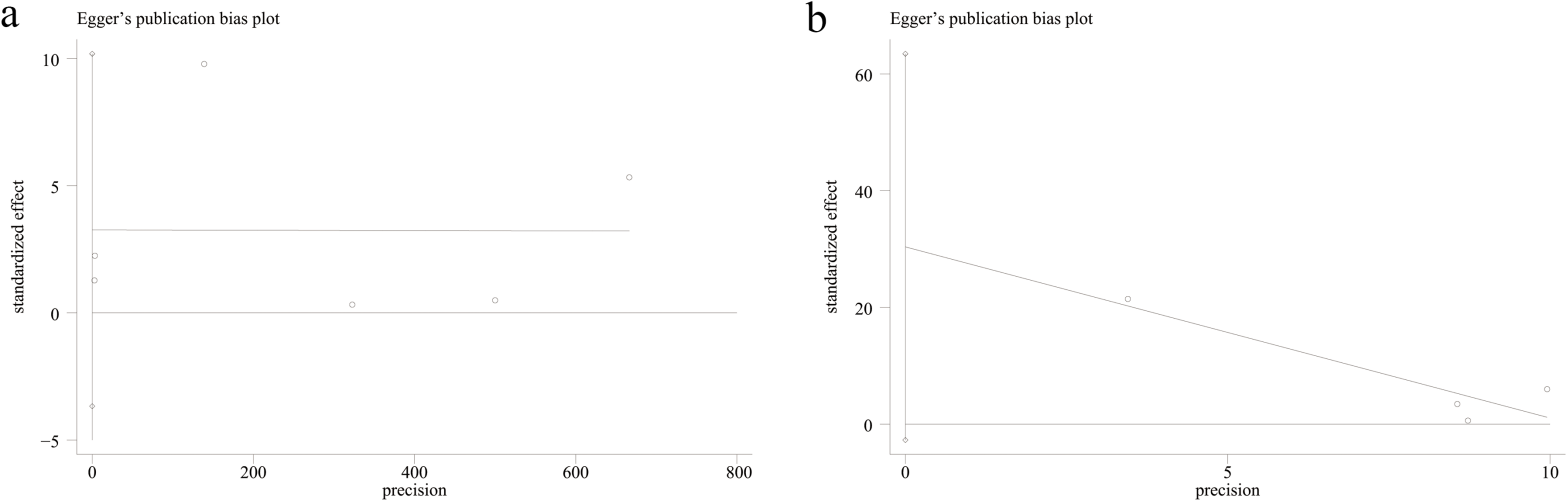
Egger’s test plot **(a)** categorical and **(b)** continuous variables.

**Figure 5:**
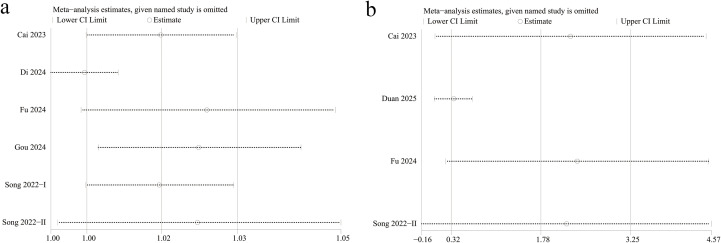
Sensitivity analysis of the **(a)** categorical and **(b)** continuous variables.

We assessed the potential publication bias through Egger’s regression tests for categorical and continuous variables. No significant publication bias was detected for either categorical (*P* = 0.261, **Figure 4A**) or continuous variables (*P* = 0.059, **Figure 4B**). In addition, we performed sensitivity analysis for the results of categorical and continuous variables to assess the effect of each study on the total OR and SMD by excluding eligible cohort studies one by one. Sensitivity analysis found that when the Di 2024 (19) and Song 2022-II (29) studies were excluded separately, the statistical differences in the categorical variable results changed from significant to non-significant (**Figure 5A**). When these two studies were excluded, heterogeneity for categorical variables decreased from 95% to 55%. In addition, when the Song 2022-II study was excluded, the statistical differences in the continuous variable results changed from significant to non-significant (**Figure 5B**).

## 4 Discussion

This study, for the first time, demonstrated through a meta-analysis that elevated PLR levels are significantly associated with an increased risk of vertebral fracture in patients with osteoporosis. This finding has profound pathophysiological underpinnings. The role of chronic low-grade inflammation in the progression of osteoporosis has become a hot topic in recent years. As a marker of systemic inflammation, elevated PLR levels reflect a pathological state characterized by increased platelet activation and lymphocyte depletion (30). Platelets play a complex dual role in bone metabolism (13). They directly promote osteoblast proliferation, differentiation, and migration, and stimulate bone matrix synthesis and angiogenesis by releasing key factors such as platelet-derived growth factor (PDGF) and transforming growth factor-β (TGF-β), which are crucial for maintaining the dynamic balance between bone formation and resorption (31, 32). For example, PDGF enhances osteoblast chemotaxis to sites of bone resorption, accelerating bone defect repair (33). Conversely, decreased TGF-β levels are closely associated with delayed bone healing and an increased risk of osteoporosis (34). On the other hand, lymphocyte subsets, particularly regulatory T cells, effectively suppress osteoclast activity and maintain bone remodeling homeostasis by producing anti-inflammatory cytokines such as interleukin-4 (IL-4) and IL-10 (35, 36). The positive correlation between PLR and vertebral fracture observed in this study provides direct clinical evidence for the theory of “chronic low-grade inflammation of the skeletal system” and supports PLR as a sensitive indicator of the inflammatory state of the bone microenvironment. Its clinical value lies in establishing a quantifiable link between systemic inflammatory responses and pathological changes in local bone tissue.

This study, using a dual data synthesis approach, revealed the strength and clinical significance of the association between PLR and vertebral fracture. In categorical analysis, the high PLR group showed a significant increase in the risk of vertebral fracture. Although the OR was close to 1, its statistical significance (P = 0.01) and narrow confidence interval indicate the robustness of this association. However, it is worth noting that the clinical effect value of categorical variable is extremely small. This highly heterogeneous and unstable association suggests that PLR has limited value as an independent predictor, and future studies need to combine it with other indicators for comprehensive evaluation. Notably, continuous analysis revealed significantly higher PLR levels in the fracture group compared with the non-fracture group. This large effect size further reinforces the pathological significance of the elevated PLR. A mean difference (SMD) of 1.78, a significant effect size, is considered a strong effect, and this study’s SMD, exceeding conventional thresholds, suggests a highly clinically significant difference in PLR between the fracture and non-fracture groups. This strength of the association may stem from the profound effects of inflammation on bone quality. Chronic inflammation not only accelerates bone resorption by promoting osteoclast activation but also inhibits key bone formation signaling pathways, such as Wnt/β-catenin, leading to a breakdown of trabecular connectivity and increased cortical porosity (37, 38). Of particular concern is that inflammatory mediators such as tumor necrosis factor-α (TNF-α) and interleukin-6 (IL-6) can directly induce osteoblast apoptosis, impairing bone’s mechanical sensing function and ultimately significantly reducing the bone’s ability to resist external impact (39, 40). From a clinical perspective, the PLR, as a derivative of a routine complete blood count, offers unique advantages: its testing cost is significantly lower than that of bone turnover markers (such as CTX and P1NP) or bone density testing, results can be obtained rapidly in an outpatient clinic, and regular follow-up is possible to track dynamic changes in inflammatory status (41). Based on the findings of this study, it is recommended that PLR be incorporated into existing fracture risk assessment systems (such as FRAX). In particular, when PLR is persistently above 180, intensive intervention measures should be initiated even if the bone density T-score is >-2.5. PLR may be particularly suitable for optimizing treatment decisions for patients with an intermediate-risk FRAX score and for the screening and management of individuals at high risk of asymptomatic vertebral fractures, providing a cost-effective and convenient risk stratification tool for clinical practice.

The high degree of heterogeneity between studies is a core point of controversy in these results, requiring further analysis of its underlying causes. Sensitivity analyses clearly revealed that the Di 2024 and Song 2022-II studies were the primary sources of heterogeneity. Excluding these two studies eliminated the statistical significance of the combined effect size for the categorical variables (P>0.05), and the heterogeneity index significantly decreased from 95% to 55%. More fundamental sources of heterogeneity are threefold. First, in terms of PLR methodology, the studies used different blood analysis systems and lacked standardized anticoagulation protocols. Second, regarding fracture assessment criteria, some studies relied on clinical symptoms or plain radiographic reports, resulting in a high rate of missed diagnoses. Studies using vertebral fracture assessment (VFA) or quantitative CT significantly improved sensitivity (42). Finally, in controlling for confounding factors, only three studies adjusted for history of glucocorticoid use—a factor that both influences PLR levels by inducing lymphopenia and independently increases fracture risk by inhibiting osteoblast function. Of particular note is the substantial variation in PLR cutoff values, reflecting the current lack of standardized reference ranges based on healthy populations. These methodological differences lead to inconsistent effect sizes across studies, highlighting the urgency of establishing a unified research framework. While the presence of heterogeneity does not negate the potential value of the PLR, it does require caution in interpreting the results and emphasizes the need for future studies to prescribe standardized testing procedures and diagnostic criteria.

Limitations of this study profoundly impact the robustness and generalizability of the conclusions. Limited sample size was the most prominent constraint: only seven observational studies were included, of which continuous variable analyses were based on only four studies, resulting in significantly underpowered statistical tests. All included studies were observational in design and subject to unavoidable residual confounding bias. For example, vitamin D deficiency was not adequately adjusted for in most studies, despite the fact that vitamin D not only regulates lymphocyte function and influences the PLR but also directly participates in bone matrix mineralization (43, 44). Fall risk, a key risk factor for fractures (45), and its potential association with the PLR were not assessed. The lack of standardized procedures for PLR measurement poses significant challenges: blood specimen processing time affects platelet aggregation, and differences in quality control standards between laboratories reduce the comparability of results. Lymphocyte differential counts are particularly susceptible to high error when using different analyzer brands. The absence of data from key subgroups limits the application of precision medicine: insufficient data are available for sex-stratified analyses, and estrogen deficiency in postmenopausal women may amplify the effects of inflammation on bone metabolism (46, 47). The absence of age-stratified analyses obscures the potential for interference with the baseline PLR by the natural decline of lymphocytes in older individuals. Furthermore, the lack of categorization by fracture type is a significant drawback: the inflammatory mechanisms underlying primary vertebral fractures and recurrent fractures may differ fundamentally, with the latter often accompanied by a persistent local inflammatory response during the healing phase. A key limitation lies in the arbitrary cutoff values for PLR diagnosis—cutoff values used in current studies are derived from statistical percentiles or receiver operating characteristic (ROC) curves, rather than pathophysiological thresholds, and do not account for adjustments for age, sex, and comorbidities. These limitations collectively prevent the current evidence from supporting the use of PLR as an independent predictor in clinical decision-making systems.

Despite methodological limitations, the translational value of PLR in osteoporosis management warrants systematic planning. Based on the positive findings of this study, a stepwise clinical application pathway is proposed: first, initiating vertebral fracture screening in routine osteoporosis clinics for patients with intermediate-risk FRAX scores and a PLR >150; second, developing a multifactorial prediction model incorporating PLR, using the FRAX-PLR score = [baseline FRAX risk] × [1 + k × (PLR - reference value)], although its calibration requires validation in prospective studies; third, exploring the value of dynamic monitoring by regularly measuring PLR in patients receiving osteoporosis treatment to assess the association between improved inflammatory status and fracture risk. Implementing this pathway requires addressing three key issues: first, establishing a PLR reference range, which is recommended to be developed using a large healthy cohort study (n >10,000) stratified by ten age groups; second, clarifying the frequency of PLR monitoring, recommending every 6–12 months based on the characteristics of the bone turnover cycle; and finally, establishing a critical intervention threshold, with consideration of adding osteoporosis medications with anti-inflammatory properties when the PLR >180. It’s important to avoid misunderstandings when using the PLR in clinical practice: transient increases in the PLR due to acute infections or tumors have no predictive value. Patients currently taking immunosuppressants or chemotherapy should interpret the results with caution. In the long term, the PLR, as a visual indicator of the “inflammation and immune imbalance in the bone microenvironment”, may offer new insights into anti-inflammatory interventions for preventing osteoporosis. For example, exploring the added value of targeted therapies such as IL-1 receptor antagonists in patients with elevated PLRs.

To overcome current research bottlenecks, a systematic research strategy is urgently needed. The primary task is to establish a globally coordinated, standardized framework for PLR research. Core elements include clearly defined testing timing, standardized anticoagulation protocols, standardized specimen handling procedures, and a cross-center quality control system. At the cohort study level, multicenter prospective observational studies should be initiated, with prespecified key subgroup analyses (stratified by sex and age, distinguishing between primary and secondary osteoporosis) and standardized imaging assessments. Further exploration is needed in the mechanistic realm: correlations between PLR components and bone turnover markers should be analyzed using flow cytometry. Quantitative relationships between PLR and bone microarchitectural parameters should be established using bone biopsy samples. Mendelian randomization studies should be conducted to validate causal associations between inflammatory markers and fractures. Of greatest translational value are interventional studies: randomizing patients with osteoporosis and a high PLR to compare the fracture prevention effects of conventional antiresorptive therapy with combined anti-inflammatory strategies. Furthermore, dynamic changes in PLR during treatment should be monitored in relation to improvements in bone density. In addition, future basic translational research needs to focus on changes in the bone marrow hematopoietic microenvironment in osteoporosis patients. This unique microenvironment is not only a habitat for hematopoietic stem cells and immune cells, but also closely related to the osteogenic-adipogenic differentiation balance of mesenchymal stem cells (48). The increased bone marrow adipose tissue, enrichment of inflammatory factors, and altered vascular function associated with osteoporosis can all reshape this microenvironment, thereby influencing immune cell function and bone remodeling cells, creating a vicious cycle (49). Exploring the correlation between PLR and specific changes in the hematopoietic microenvironment may be an important bridge connecting systemic inflammatory markers and local bone pathogenesis mechanisms.

## 5 Conclusion

This study is the first to confirm the positive association between elevated PLR and the risk of osteoporotic vertebral fractures using evidence-based medicine methods, providing quantitative evidence for the role of inflammatory mechanisms in bone metabolic diseases. Despite significant heterogeneity and methodological limitations, PLR still demonstrates translational potential as an economical and convenient biomarker. Researchers urgently need to prioritize the standardization of PLR and improve risk stratification models using large prospective cohorts. Medical decision-makers should pay attention to the unique value of inflammatory markers in fracture prevention and explore innovative solutions to incorporate PLR into osteoporosis diagnosis and treatment pathways.

## Data Availability

The original contributions presented in the study are included in the article/**Supplementary Material**. Further inquiries can be directed to the corresponding author.
