# Supplementary material for: Association between platelet to lymphocyte ratio and the risk of vertebral fracture in patients with osteoporosis: a systematic review and meta-analysis
Source: Front Endocrinol (Lausanne). 2026 Mar 25;17:1705468. doi: 10.3389/fendo.2026.1705468 (PMC13056836; doi:10.3389/fendo.2026.1705468)
Supplement: Supplementary file 1 [file DataSheet1.docx]

**TableS1** Literature search strategy

Pubmed-7

((((("Blood Platelets"[Mesh]) OR (((((Blood Platelet) OR (Platelets)) OR (Platelet)) OR (Thrombocytes)) OR (Thrombocyte))) AND (("Lymphocytes"[Mesh]) OR (((Lymphocyte) OR (Lymphoid Cells)) OR (Lymphoid Cell)))) AND (Ratio)) AND (("Fractures, Bone"[Mesh]) OR (Fracture))) AND (("Osteoporosis"[Mesh]) OR (Osteoporoses))

Embase-25

((Blood Platelets or (Blood Platelet or Platelets or Platelet or Thrombocytes or Thrombocyte)) and (Lymphocytes or (Lymphocyte or Lymphoid Cells or Lymphoid Cell)) and Ratio and (Fractures, Bone or Fracture) and (Osteoporosis or Osteoporoses)).af.

Cochrane-0

((Blood Platelets or (Blood Platelet or Platelets or Platelet or Thrombocytes or Thrombocyte)) and (Lymphocytes or (Lymphocyte or Lymphoid Cells or Lymphoid Cell)) and Ratio and (Fractures, Bone or Fracture) and (Osteoporosis or Osteoporoses)).af.

Web of Science-15

(((((Blood Platelets) OR (((((Blood Platelet) OR (Platelets)) OR (Platelet)) OR (Thrombocytes)) OR (Thrombocyte))) AND ((Lymphocytes) OR (((Lymphocyte) OR (Lymphoid Cells)) OR (Lymphoid Cell)))) AND (Ratio)) AND ((Fractures, Bone) OR (Fracture))) AND ((Osteoporosis) OR (Osteoporoses)) (Topic)

Wanfang-9

全部:(血小板) and 全部:(淋巴细胞) and 全部:(比值 OR 比例) and 全部:(骨质疏松) and 全部:(骨折)

CNKI-9

篇关摘:血小板(模糊))AND(篇关摘:淋巴细胞(模糊))AND(篇关摘:比值 + 比例(模糊))AND(篇关摘:骨质疏松(模糊))AND(篇关摘:骨折(模糊))
